# Discovery of Four Novel Viruses Associated with Flower Yellowing Disease of Green Sichuan Pepper (*Zanthoxylum armatum*) by Virome Analysis

**DOI:** 10.3390/v11080696

**Published:** 2019-07-31

**Authors:** Mengji Cao, Song Zhang, Min Li, Yingjie Liu, Peng Dong, Shanrong Li, Mi Kuang, Ruhui Li, Yan Zhou

**Affiliations:** 1 National Citrus Engineering Research Center, Citrus Research Institute, Southwest University, Chongqing 400712, China; 2 Academy of Agricultural Sciences, Southwest University, Chongqing 400715, China; 3 Chongqing Agricultural Technology Extension Station, Chongqing 401121, China; 4 USDA-ARS, National Germplasm Resources Laboratory, Beltsville, MD 20705, USA

**Keywords:** RNA viruses, *nepovirus*, *idaeovirus*, *enamovirus*, *nucleorhabdovirus*, high-throughput sequencing, transcriptome, small RNA, RNA silencing, RT-PCR

## Abstract

An emerging virus-like flower yellowing disease (FYD) of green Sichuan pepper (*Zanthoxylum armatum* v. *novemfolius*) has been recently reported. Four new RNA viruses were discovered in the FYD-affected plant by the virome analysis using high-throughput sequencing of transcriptome and small RNAs. The complete genomes were determined, and based on the sequence and phylogenetic analysis, they are considered to be new members of the genera *Nepovirus* (*Secoviridae*), *Idaeovirus* (unassigned), *Enamovirus* (*Luteoviridae*), and *Nucleorhabdovirus* (*Rhabdoviridae*), respectively. Therefore, the tentative names corresponding to these viruses are green Sichuan pepper-nepovirus (GSPNeV), -idaeovirus (GSPIV), -enamovirus (GSPEV), and -nucleorhabdovirus (GSPNuV). The viral population analysis showed that GSPNeV and GSPIV were dominant in the virome. The small RNA profiles of these viruses are in accordance with the typical virus-plant interaction model for *Arabidopsis thaliana*. Rapid and sensitive RT-PCR assays were developed for viral detection, and used to access the geographical distributions. The results revealed a correlation between GSPNeV and the FYD. The viruses pose potential threats to the normal production of green Sichuan pepper in the affected areas due to their natural transmission and wide spread in fields. Collectively, our results provide useful information regarding taxonomy, transmission and pathogenicity of the viruses as well as management of the FYD.

## 1. Introduction

**Figure 1:**
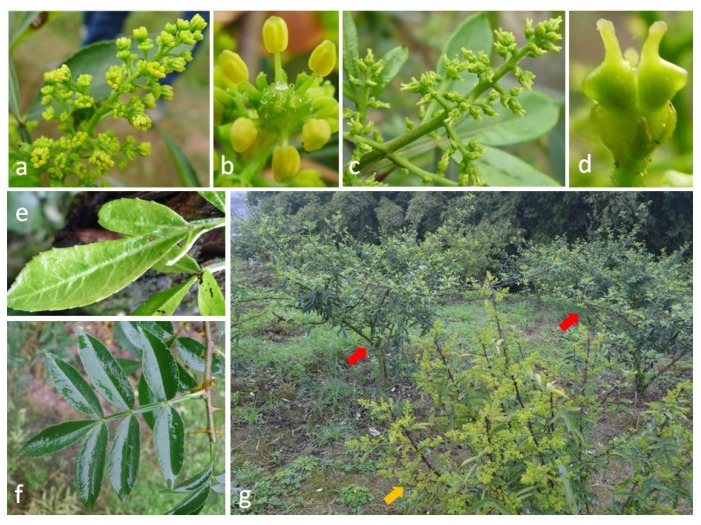
(**a**,**b**,**e**) Green Sichuan pepper plant infected with flower yellowing disease (FYD) exhibiting yellowing and tumescent stamen, and yellowing and small leaf. (**c**,**d**,**f**) Normal stamen and leaf of healthy plant. (**g**) The FYD-affected tree (yellow arrow) among asymptomatic trees (red arrows).

*Zanthoxylum* is a genus of approximately 225 species of deciduous and ever green shrubs, trees and woody climbers in the family *Rutaceae* [1]. It is native primarily to tropical and subtropical regions, and some of its species extend to warm temperate zones [2,3]. The genus is economically important and has been used in pharmaceutics, cosmetics, and culinary applications [2,3,4]. Leaves, bark, fruits and fruit oil are utilized in traditional medicines to treat asthma, common cold, gout, jaundice, diarrhea, pain and stomach upsets [4,5,6]. Fruits and fruit oil are also extensively used as spice in eastern Asian countries [3,6]. In China, for a long history of cultivation the production of *Zanthoxylum* is so far worth multi-billion dollars. In particular, green Sichuan pepper (*Z. armatum* v. *novemfolius*) is one of the most economically important commercial crops in Chongqing Province, which produces 70% of dry fruits of the variety nationwide. For instance, in 2018, approximately 530,000 acres of this variety were planted only in Jiangjin District, with a record high sale value of over half billion dollars. Several pathogens including fungi and phytoplasmas cause serious diseases in cultivated *Zanthoxylum* spp. [7,8,9,10,11]. However, no virus or viroid has previously been reported to be associated with this crop. Recently, a virus-like disease, flower yellowing disease (FYD), was found on green Sichuan pepper trees in Chongqing. Typical symptoms observed of the FYD include internode shortening, leaf curl, pistil abortion, stamen yellowing and intumescence, and eventually fruit dropping. After an irreversible and degenerative progress during a few years, the diseased trees decline and die, thus causing severe yield reduction in crop production. Initial symptoms of the blossoms usually appear only on a few branches of diseased trees, and more and more branches show the symptoms following the disease progress. With propagations of green Sichuan pepper by seeds and grafting, symptomatic trees were randomly distributed in the fields (Figure 1g). It appears that the FYD is naturally transmitted someway from source trees to healthy trees. Unfortunately, the etiology of the disease is unknown.

Advances in high-throughput sequencing (HTS) have led to the development of metagenomic analyses of genomes from a variety of living forms [12]. This technology has also been used to study populations of many organisms. One such example is viral metagenomics or the generation of the virome from a single species, instead of entire microbial communities [13]. The first study of human virome was initiated as a systematic exploration of viruses infecting humans [14]. This systematic approach for viruses was then extended to animals and plants [15,16]. The study of plant viromes such as that of grapevine and pepper provided significant information on viral populations, and viral quasispecies [17,18]. In principle, HTS techniques such as RNA sequencing (RNA-seq) and small RNA sequencing (sRNA-seq) yield short sequences of viral RNA or sRNA entity extracted from plants for in silico assembly to generate longer sequences (contigs) that facilitate BLAST-based or BLAST-independent annotation [19].

As for this study, the HTS of both RNA- and sRNA-seq coupled with the bioinformatics analysis allowed discovery of four new RNA viruses, tentative members of the genera *Nepovirus* (subgroup C), *Idaeovirus*, *Enamovirus* and *Nucleorhabdovirus*, in diseased green Sichuan pepper plants. To understand the cause of FYD, a subsequent large-scale field survey, during which many indications pointed to the nepovirus, was performed. The prevalence of viruses and FYD as well as associated symptoms, and the fact which demonstrated that the viruses have the ability to spread through farm operation (grafting), or still unspecified natural transmissions in the open field, could not be neglected so that relevant works are of great significance.

## 2. Materials and Methods

### 2.1. Plant Materials

In June 2017, leaves and inflorescences were collected from green Sichuan pepper trees with or without symptoms and stored at −80 °C. One tree with foliar yellowing and dwarf as well as flower yellowing and deformation was used for high-throughput sequencing (HTS) analysis (Figure 1a,b,e). An asymptomatic tree was used as a negative control (Figure 1c,d,f).

### 2.2. RNA Preparation and HTS

To identify potential viral and viroidal pathogens, total RNA was extracted from both samples using the EASY spin Plus Complex Plant RNA Kit (Aidlab, China) for RNA sequencing (RNA-seq), and total sRNA was extracted from the samples using the EASYspin Plant microRNA Extract kit (Aidlab) for small RNA sequencing (sRNA-seq). For RNA-seq, after depletion of the ribosomal RNA, the libraries were constructed with a TruSeq RNA Sample Prep kit (Illumina, USA) and sequenced using the Illumina HiSeq X-ten platform (pair-end reads of 150 bp). The sRNA libraries were built using a TruSeq Small RNA Sample Prep Kit (Illumina) and sequenced using the Illumina Hiseq2500 platform (1 × 50 bp read lengths).

### 2.3. HTS Data Analysis and Virus Identification

RNA reads obtained from RNA- and sRNA-seq were analyzed using the software CLC Genomics Workbench 9.5 (Qiagen, Germany). After adaptor trimming and filtering of raw reads, *de novo* assembly of clean reads was carried out using the algorithms Trinity (RNA) or Velvet (sRNA) [20,21]. The clean reads from RNA-seq were mapped against the *Citrus sinensis* and *C*. *clementina* reference genomes (downloaded from https://www.citrusgenomedb.org) to remove host reads and then assembled, while sRNA clean reads being trimmed from sRNA-seq were directly assembled. Assembled contigs were subjected to a BLASTx and BLASTn search (https://blast.ncbi.nlm.nih.gov/Blast.cgi) of viral (taxid:10239) and viroidal (taxid:12884) sequences in GenBank Databases, respectively.

### 2.4. Determination of Virus Genomes

To determine the genome sequence of a novel virus, specific primers (Table S1) were designed based on its contig sequences obtained in this study and used in RT-PCR for amplification of overlapped viral fragments. One-step RT-PCR was carried out using the PrimeScript One Step RT-PCR Kit (Takara, Japan). RACE (5′ and 3′ termini) was performed using the GeneRacer Core Kit (Invitrogen, USA). PCR amplicons were purified using the Gel Extraction Kit (Biomega, USA) and then cloned using the pEASY-T1 Vector System (TransGen, China). At least five clones of each PCR amplicon were fully sequenced (Sanger), and sequences of all amplicons were assembled to generate complete genome sequences. 

### 2.5. HTS Data Reanalysis

The resulted full viral genome sequences were used as references to map against both RNA-seq and sRNA-seq clean reads by the CLC Genomics. Average coverage was calculated by multiplying the number of mapped reads by the average read length (149.74 nt for RNA-seq; 21.24 nt for sRNA-seq) and dividing the total by the length of the genome. In addition, small RNAs of each virus were categorized according to the genome polarity, size and 5’ end nucleotide preference, analyzed with statistics and visualized.

### 2.6. Sequence and Phylogenetic Analysis

Standard procedure for the genome sequence analysis of a new virus was as follows: (I) Open reading frame (ORF) of the viral genome or genome segment was identified by the NCBI ORF finder (https://www.ncbi.nlm.nih.gov/orffinder); (II) closely related viruses were found by the BLASTp search of the databases using amino acid (aa) sequences of all ORF-encoded proteins; (III) protein molecular weight was estimated using the CLC Genomics; (IV) conserved domain of putative protein was detected by the Conserved Domain Database (CDD) of NCBI (https://www.ncbi.nlm.nih.gov/Structure/cdd/wrpsb.cgi); (V) conserved motif of putative protein was found by the multiple sequence alignment of new virus and related viruses using the ClustalW [22].

Phylogenetic relationships between each novel virus and a range of related viruses were analyzed by Mega 7 [23], using amino acid sequences of entire or partial proteins, which are conserved, and commonly selected for phylogenetic reconstruction of these viruses. All the maximum-likelihood trees were generated with the MUSCLE sequence alignment, Jones-Taylor-Thornton (JTT) model, and gaps complete deletion treatment, and 1000 bootstrap replicates applied.

### 2.7. RT-PCR Protocols and Field Surveys

One-step RT-PCR assays using the PrimeScript One Step RT-PCR Kit (Takara, Japan) were developed for the detection of the four viruses, one for each virus, and used to test the field samples collected earlier. Virus specific primer sequences, reaction system and thermocycling condition for each virus are listed in Tables S1 and S2.

To investigate the distribution and infection rates of the four viruses in main green Sichuan pepper growing areas in Chongqing, a total of 217 leaf samples were tested. 186 leaf samples were collected from FYD-infected (41) and asymptomatic (145) trees in fields. The sampled areas included Fulu, Guangpu and Sanhe of Bishan County, Xianfeng and Ciyun of Jiangjin District, Shichuan of Yubei District and Dandu of the Changshou District. Most of these areas are adjacent to the Jiangjin District where the sequencing samples were initially collected.

For the purpose of finding evidence of potential natural virus transmission, leaf samples were tested from 31 seedlings from an open field of the Ciyun breeding base where FYD is common in Chongqing.

Furthermore, to analyze the presence of GSPNeV in symptomatic and asymptomatic branches of the same tree, both symptomatic and asymptomatic branches of four GSPNeV positive plants were separately tested by RT-PCR.

### 2.8. Graft Transmission

ZP-9, a tree with flower yellowing symptoms, was confirmed to be naturally infected with all four viruses by RT-PCR. Mature branches were collected from the ZP-9 and a healthy tree. To prove that these viruses are active entities rather than fragments integrated into the host genome, green bark chips of each source were separately grafted onto five virus-free green Sichuan pepper seedlings as stocks, with three bark inoculations per stock. Additionally, to support that GSPNeV is transmissible, buds from a symptomatic tree infected only with this virus were also grafted onto five stocks in a similar way. The grafted seedlings were placed in a growth chamber with 12-hr light/12-hr dark, temperature at 25 °C and relative humidity at 75%.

## 3. Results

### 3.1. Virus Discovery by Homology Analysis

Total clean reads of 58,077,354 (RNA-seq) and 29,274,944 (sRNA-seq) were obtained for the symptomatic tree. From the asymptomatic sample, total clean reads of 82,273,416 (RNA-seq) and 25,309,295 (sRNA-seq) were obtained. A total of 23,010,248 and 17,537,997 RNA-seq clean reads were retained for the symptomatic and asymptomatic samples, respectively, after removal of the total reads with 60% or higher similarities to the *Citrus* spp. reference genomes. *De novo* assembly of the unmapped RNA-seq reads and clean sRNA-seq reads (mapping to the *Citrus* spp. reference genomes was skipped) yielded 126,144 RNA-seq contigs and 6,702 sRNA-seq contigs for the symptomatic sample. For the asymptomatic sample, 164,598 and 6,528 contigs were generated from RNA- and sRNA-seq, respectively. The BLASTx search of the NCBI databases revealed the presence of six contigs with moderate similarities to four known viruses belonging to different plant virus genera among the RNA-seq contigs of the symptomatic sample (Table 1). More viral contigs of the same virus types were detected from the sRNA-seq contigs of the FYD sample, but the length of the contigs was much shorter (Table 1). These viral contigs were not identified from either the RNA-seq or sRNA-seq data of the asymptomatic sample, suggesting that the four novel viruses are strongly associated to FYD. With respect to the aa sequence divergence (>25%) of each of the viral contigs from the most closely related known virus, thus the four novel viruses were provisionally named as green Sichuan pepper nepovirus (GSPNeV), -idaeovirus (GSPIV), -enamovirus (GSPEV), and -nucleorhabdovirus (GSPNuV). No viriod was detected in either the FYD or asymptomatic sample.

Complete genome sequences of all four viruses were reconstructed from molecular cloning and sequencing analysis, and submitted to GenBank under accession numbers MH323435 (RNA1) and MH323434 (RNA2) for GSPNeV; MH323432 (RNA1) and MH323433 (RNA2) for GSPIV; MH323437 for GSPEV and MH323436 for GSPNuV.

### 3.2. Genome Characterization and Classification of GSPNeV

**Figure 2:**
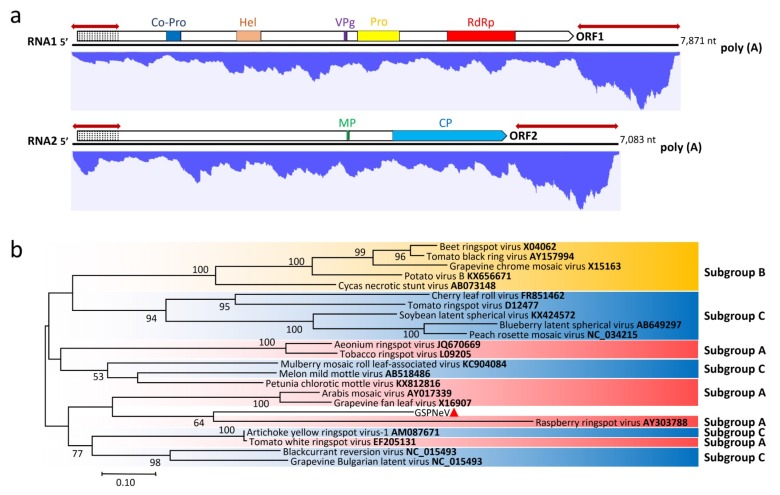
(**a**) Genomic structure of green Sichuan pepper-nepovirus (GSPNeV) and distribution profile of transcriptomic reads from GSPNeV. The colorful blocks in putative proteins represent conserved amino acid domains or motifs. Co-Pro: Proteinase co-factor. Hel: Helicase. VPg: Viral protein linked to the genome. Pro: Proteinase. RdRp: RNA dependent RNA polymerase. Poly (A): A 3′ terminal poly (A) tail. Similar-sized double-headed red arrows indicate extensive regions of identical nucleotides between RNA1 and RNA2; dotted areas at 5′ end of coding region indicate extensive region of identical amino acids; (**b**) maximum-likelihood relationships [Jones-Taylor-Thornton (JTT) model] inferred from amino acid sequence alignments of the conserved CG-GDD region between Pro and RdRp domains of GSPNeV (marked with small red triangle) and representative nepoviruses divided into three subgroups (A, B, and C). Bootstrap support (as a percentage of 1000 replicates) is shown at the nodes for values greater than 50%. Accession numbers are supplied in taxa names.

The GSPNeV genome comprises RNA1 of 7082 nt and RNA2 of 7083 nt, respectively, excluding the poly (A) tail. Each RNA contains one open reading frame (ORF). The 5′ untranslated regions (UTR) of RNA1 and RNA2 are both 54 nt, and the 3′ UTRs are 1358 nt for RNA1 and 1443 nt for RNA2. Highly conserved nucleotide sequences were found in both termini of the two RNAs of GSPNeV (Figure 2a), suggesting it might belong to subgroup C of *Nepovirus* [24]. The sequence of the 5’ terminus is 615 nt, consisting of the 5’ UTRs and 5’ partial sequence of the coding region. The 3’ terminal sequence is 1334 nt located in the 3’ UTRs. The sequence identities between RNA1 and RNA2 are 71.6% for the 5’ conserved region and 99.0% for the 3’ conserved region. ORF1 (nt 55–6513) of RNA1 encodes a putative polyprotein of 2152 aa with a predicted molecular weight of approximately 241.8 kDa. It contains conserved domains of proteinase co-factor (Co-pro; motifs F_387_-W_415_-E_451_), helicase (Hel; motifs G_696_-G_701_KS-D_746_D), viral genome-linked protein (VPg; conserved aa D_1157_-Y_1161_-R_1164_N-R_1171_), protease (Pro; motifs H_1217_-E_1267_-C_1373_G-G_1393_-G_1398_) and RNA-dependent RNA polymerase (RdRp; motifs D_1706_-D_1711_-G_1769_-T_1773_-N_1777_-G_1811_DD) (Figure 2a) [25,26]. Interestingly, aa sequence alignments showed that the crucial aa Val_1395_ in the substrate binding pocket of the potential catalytic domain of the protease was substituted by His or Leu in GSPNeV-Pro. RNA2 contains one ORF (nt 55–5640) encoding a putative polyprotein of 1822 aa with an estimated molecular mass of 207.6 kDa. It consists of movement protein (MP; motifs A_1169_PL) and coat protein (CP; motifs F_1853_YGN), which are slightly different from motifs LPL (MP) and FYGR (CP) of other nepoviruses (Figure 2a) [27,28].

Sequence comparisons of GSPNeV and other nepoviruses showed that the virus had the highest aa sequence identities with grapevine Bulgarian latent virus (GBLV, subgroup C) at the RNA1 polyprotein (29.9%) and blueberry latent spherical virus (BLSV; subgroup C) at the RNA2 polyprotein (21.0%). The highest aa sequence identities were 42.4% at the CG-GDD region (between Pro motif CG and RdRp motif GDD) between the GSPNeV and petunia chlorotic mottle virus (PCMV, subgroup A) and 23.2% at the CP region between the GSPNeV and mulberry mosaic roll leaf-associated virus (MMLRaV, subgroup A). These values are much lower than those used for species demarcation criteria of 80% at the GC-GDD region and 75% at the CP region for the family *Secoviridae* [24]. The phylogenetic analysis placed GSPNeV and other known nepoviruses of subgroup A and C in the same cluster, supporting GSPNeV as a nepovirus of subgroup C (Figure 2b) based on similar genome structures shared within them. Thus, GSPNeV should be considered as a new species of the genus *Nepovirus*.

### 3.3. Genome Characterization and Classification of GSPIV

**Figure 3:**
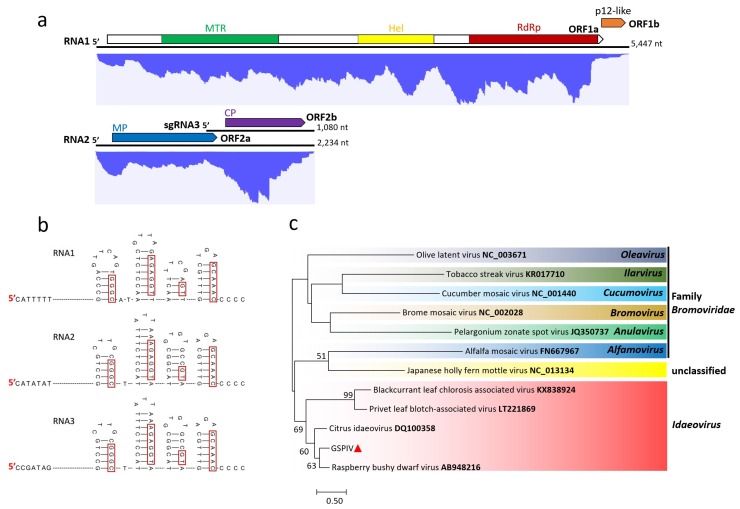
(**a**) Genomic structure of green Sichuan pepper-idaeovirus (GSPIV) and distribution profile of transcriptomic reads from GSPIV. The colorful blocks in proteins represent conserved amino acid sequences, domains or motifs. MTR: Methyltransferase. Hel: Helicase. RdRp: RNA dependent RNA polymerase. p12-like: Putative p12-like protein. MP: Movement protein. CP: Coat protein. sgRNA3: Subgenomic RNA3; (**b**) first seven nucleotides of 5′ genomic terminus and, stem-loops and the last conserved five cytidines (C) of 3′ genomic terminus from RNA1, 2 and sgRNA3 are shown. The identical nucleotides at the stem shared among the RNAs are indicated with red boxes; (**c**) maximum-likelihood tree (JTT model) derived from entire CP amino acid sequence comparisons of GSPIV (marked with small red triangle), representative idaeoviruses, and related viruses. Bootstrap support values (as a percentage of 1000 replicates) are shown at the nodes if greater than 50%. The accession number is given in taxa names.

The GSPIV genome consists of two genomic RNAs, a 5447-nt RNA1 and a 2234-nt RNA2, and an additional 1080-nt RNA2-derived subgenomic (sg) named sgRNA3 (Figure 3a). All RNAs have similar 3’-terminal stem-loop structures and repeated cytidines (C) as reported for idaeoviruses (Figure 3b), but lack the conserved hexanucleotide in their 5′ terminus [29]. It is noticed that the 5′ terminus begins with a C, instead of the adenine (A) of the known idaeoviruses (Figure 3b) [30,31]. The unusual C added at a positive-strand RNA viral 5′ terminus has been previously found [32]. RNA1 consists of a 95-nt UTR, two overlapping ORFs and a 50-nt 3’ UTR. ORF1a (nt 96–5186) and ORF1b (nt 5146–5397) are predicted to encode a polyprotein (1696 aa, 192.2 kDa) and a p12-like protein (83 aa, 9.6 kDa), respectively. Three conserved domains, methyltransferase (MTR; aa 185–585), Hel (aa 857–1115) and RdRp (aa 1236–1676), were found in the polyprotein, indicating it is a putative viral replicase. The p12-like protein of unknown function contains a transmembrane (TM) domain (aa 20–39) [33]. The 5′ UTR and 3′ UTR of RNA2 are 142 nt and 83 nt, respectively. Similar to the raspberry bushy dwarf virus (RBDV) and citrus idaeovirus of genus *Idaeovirus*, the GSPIV RNA2 has a long intergenic region (IR) of 95 nt. The two putative proteins encoded by ORF2a (nt 143–1237) and ORF2b (nt 1333–2151) are MP (364 aa, 39.6 kDa) and CP (272 aa, 30.58 kDa), respectively.

The RNA1 polyprotein of GSPIV has aa sequence identities of 71.6% with RBDV, 47.3% with black currant leaf chlorosis associated virus (BCLCaV; genus *Idaeovirus*) and 47.2% with privet leaf blotch-associated virus (PrLBaV; genus *Idaeovirus*). Its p12-like protein is 31.5% and 13.1% identical to that of RBDV and Japanese holly fern mottle virus (JHFMoV; unassigned species). The aa sequence identities between GSPIV and other known idaeoviruses range from 19.0% (PrLBaV) to 65.1% (RBDV) in the MP and 24.7% (BCLCaV) to 72.8% (RBDV) in the CP. The maximum-likelihood tree constructed from the CP sequences of GSPIV and other known idaeoviruses groups them in a distinct cluster (Figure 3c). The genome organization, sequence similarities and phylogenetic analysis all suggest that GSPIV should be considered as a new idaeovirus, thus representing the fifth idaeovirus described.

### 3.4. Genome Characterization and Classification of GSPEV

**Figure 4:**
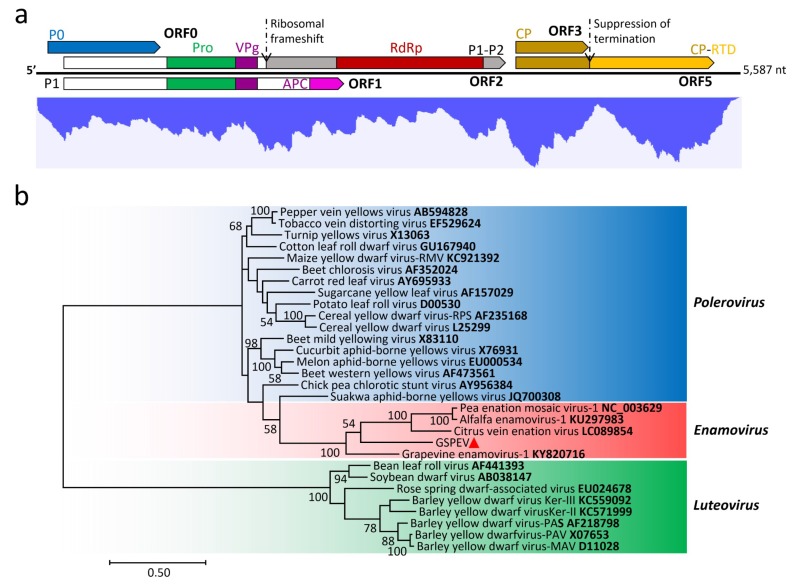
(**a**) Genomic structure of green Sichuan pepper-enamovirus (GSPEV) and profile distribution of transcriptomic reads from GSPEV. The colorful blocks in proteins represent conserved amino acid sequences, domains or motifs. P0, P1 and P1-P2 are the protein names. Pro: Proteinase. Hel: Helicase. VPg: Viral protein linked to the genome. RdRp: RNA dependent RNA polymerase. APC: APC domain. CP: Coat protein. CP-RTD: CP-RTD fusion protein; (**b**) phylogenetic tree constructed from amino acid sequences of the RdRp domains within GSPEV (marked with small red triangle) and viruses of the family *Luteoviridae*, using the maximum-likelihood method with JTT model, 1000 bootstrap replicates, and with the bootstrap percent values >50 shown on the nodes. The accession number associated with a virus is in taxa names.

The GSPEV genome of 5587 nt is the smallest among enamoviruses. Its genome organization is typical of enamoviruses, consisting of five ORFs (Figure 4a). GSPEV has a 5′ UTR (90 nt) shorter than that of other enamoviruses (180–334 nt), but the size of its 3′ UTR (211 nt) is similar to that of others (201 nt to 315 nt). ORF0 (nt 91–993) encodes a putative P0 (300 aa, 33.2 kDa), which potentially functions as an RNA silencing suppressor [34]. ORF1 (nt 218–2446) partially overlaps ORF0 and encodes putative P1 of 742 aa (81.1 kDa) by means of ribosomal leaky scanning. P1 has a 3C-like serine peptidase domain (aa 273–455) and a potential APC basic domain (aa 650–739) [35]. Alignment of the P1 aa sequences of GSPEV and other enamoviruses revealed features related to VPg, namely the upstream aa cleavage site E_454_/S and downstream conserved W_513_AD motif followed by a D/E-rich region [36]. Translation of the P1-P2 fusion protein (1168 aa, 129.7 kDa) by ORF2 (nt 1819–3723) is possibly guided by viral transcription of a G_1819_GGAAAC_1825_ signal and a potential pseudoknot (nt 1833–1851) with -1 ribosomal frameshift from ORF1 [37]. The fusion protein contains an RdRp domain (nt 721–1106). ORF3 (nt 3802–4395) encodes a putative CP protein of 197 aa (21.4 kDa). ORF5 (nt 4396–5376), with suppression of ORF3 termination, is responsible for expressing a putative CP-RTD fusion protein (524 aa, 57.0 kDa), which contains a readthrough protein domain (aa 227–513) and is essential for aphid transmission [38]. The C-rich CCNNNN tandem repeat sequences (nt 4408–4463) associated with the readthrough mechanism are located at downstream of the readthrough site A_4390_AA**UAG**GAG_4398_ [39]. 

GSPEV is most closely related to grape vein enamovirus (GEV), and the aa sequence identities of the individual proteins between them range from 22.7% (P1) to 43.7% (CP-RTD). The phylogenetic analysis derived from the RdRp aa sequences grouped GSPEV and GEV in a branch separated from other three enamoviruses in the enamovirus cluster (Figure 4b). Given these features, GSPEV should be considered as a divergent new enamovirus, the tentative fifth member of the genus.

### 3.5. Genome Characterization and Classification of GSPNuV

**Figure 5:**
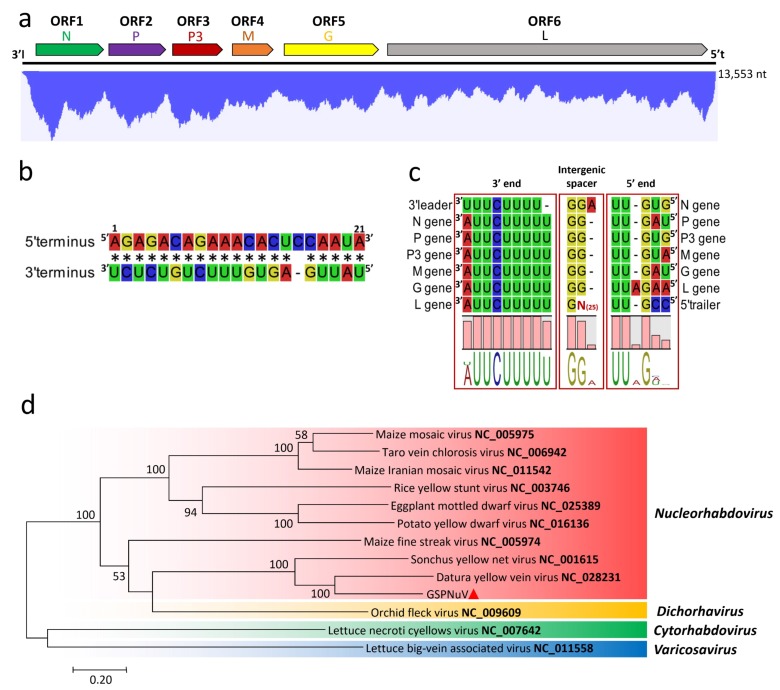
(**a**) Genomic structure of green Sichuan pepper-nucleorhabdovirus (GSPNuV) and profile distribution of transcriptomic reads from GSPNuV. N, P, P3, M, G, and L are putative nucleocapsid protein, phosphoprotein, movement protein, matrix protein, glycoprotein, and polymerase, respectively. 3’l and 5’t are a leader sequence at 3′ genomic terminus and a trailer sequence at the 5′ terminus, respectively; (**b**) the complementary sequences of the 5′ and 3′ termini (asterisk indicates complementary bases); (**c**) conserved gene junction sequences interposed between two adjacent genes; (**d**) phylogenetic tree generated using the maximum-likelihood method (JTT model) and 1000 bootstrap replicates, based on alignment of amino acid sequences of the L genes from GSPNuV (marked with small red triangle) and related viruses. Bootstrap percent values greater than 50 are shown at the nodes. The viral accession number is marked in bold in taxa names.

The complete genome of GSPNuV is 13,553 nt long and contains six ORFs in negative polarity (Figure 5a). The 3’ leader (l) sequence of 160 nt and 5’ trailer (t) sequence of 102 nt share 20/22 complementary nucleotides at their termini, forming a putative handle structure for replication (Figure 5b). The terminal 7 nt of both l and t sequences are identical to that of the sonchus yellow net virus (SYNV, genus *Nucleorhabdovirus*). The conserved gene junction sequence (3’-AUUCUUUUUGGUUGHN-5’) is almost identical to that of SYNV and datura yellow vein virus (DYVV, genus *Nucleorhabdovirus*) (Figure 5c) [40,41]. Six putative proteins encoded by ORF1 to ORF6 on the GSPNuV genome are arranged in order of nucleocapsid protein (N; 460 aa, 51.6 kDa), phosphoprotein (P; 360 aa, 40.7 kDa), MP (P3; 332 aa, 37.5 kDa), matrix protein (M; 267 aa, 29.8 kDa), glycoprotein (G; 635 aa, 71.8 kDa) and polymerase (L; 2118 aa, 241.3 kDa) (Figure 5a) [42]. The polymerase was predicted to contain domains of RdRp (aa 236–1132) and mRNA-capping region V (aa 1150–1375), with the latter being essential to mRNA cap formation [43].

GSPNuV is most closely related to DYVV, and they shared the highest aa sequence identities of 68.0% at N, 33.4% at P, 46.7% at P3, 37.9% at M, 49.0% at G and 49.0% at L. A phylogenetic tree based on the polymerase (L) aa sequences of GSPNuV, other nucleorhabdoviruses and selected rhabdoviruses placed GSPNuV with DYYV and the sonchus yellow net virus in a subgroup distinct from other nucleorhabdoviruses (Figure 5d). Considering the genome organization, the protein sequences, the phylogenetic relationships, and the host range, we propose GSPNuV as a new member of the genus *Nucleorhabdovirus* in the family *Rhabdoviridae*.

### 3.6. Transcriptomic Analysis

The RNA-seq clean RNA reads were mapped to Sanger-sequencing generated viral genomes (Figure 2a, Figure 3a, Figure 4a and Figure 5a). In general, the mapped reads were more numerous in the coding regions than UTRs and intergenic regions of the genomes, especially for GSPNuV (Figure 5a). In the virome, the GSPNeV reads were the most abundant, followed by GSPIV, GSPEV and GSPNuV (Table 1). Copy number (average coverage) computations showed that GSPIV-RNA2 was the highest, followed by GSPNeV, GSPIV-RNA1, GSPEV and GSPNuV (Table 1). Thus, viral populations of GSPNeV and GSPIV appear to be higher than those of GSPEV and GSPNuV in the FYD-affected tree.

### 3.7. Small RNA Analysis

**Figure 6:**
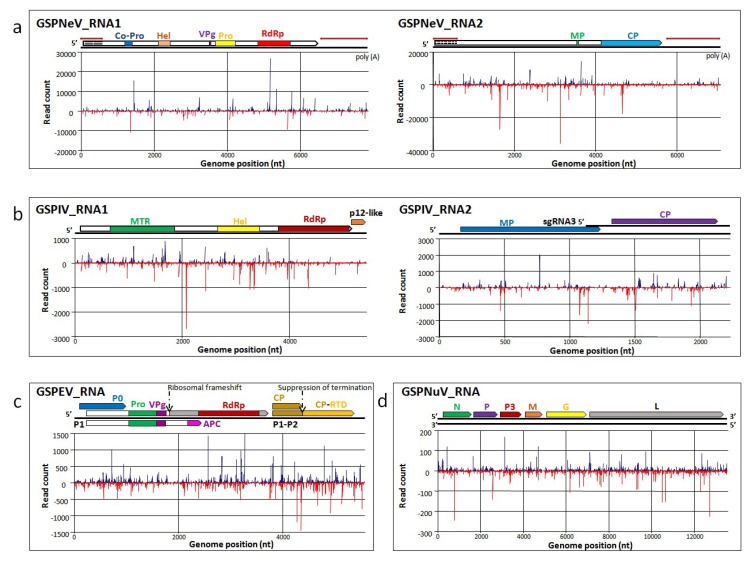
The vsiRNA read distribution across the genome of GSPNeV (**a**), GSPIV (**b**), GSPEV (**c**) and GSPNuV (**d**); the blue lines above the genome and red lines beneath the genome represent mapped genomic and anti-genomic vsiRNAs, respectively.

**Figure 7:**
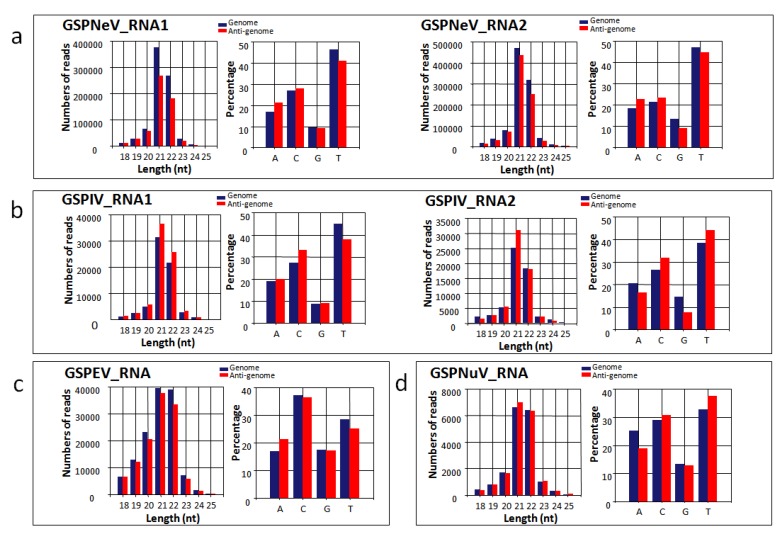
The vsiRNA size distribution, and 5′ end nucleotide preference of GSPNeV (**a**), GSPIV (**b**), GSPEV (**c**), and GSPNuV (**d**).

The sRNA-seq reads of both green Sichuan pepper samples ranged from 18 nt to 25 nt and are highly concentrated in 21 nt, 22 nt and 24 nt lengths (Figure S1). The mapping results showed that mapped vsiRNAs (virus-derived small interfering RNAs) spread evenly along the genome and anti-genome and, sometimes, formed hotspots in the ORF regions (Figure 6). High accumulation of 21 nt and 22 nt vsiRNAs (Figure 7) indicated that RNA silencing in green Sichuan pepper has a mechanism operating with (DCL) homologs (DCL4 and DCL2), similar to that of *Arabidopsis thaliana* [44]. According to the 5′ nucleotide preference of vsiRNAs (21 nt, 22 nt; dominant U) derived from GSPNeV, GSPIV, and GSPNuV (Figure 7), green Sichuan pepper argonaute (Ago) protein homologs involved with these viruses could be Ago1. In contrast, the dominant C of GSPEV vsiRNAs suggests the involvement of Ago5 homologs in green Sichuan pepper [45]. The vsiRNAs of GSPNeV constitute a higher percentage of total sRNA-seq reads than those of GSPIV, GSPEV, and GSPNuV (Table 1). The average coverage numbers calculated from GSPNeV vsiRNAs are also the highest (Table 1). It is possible that due to a more active interaction with the host, GSPNeV induced more vsiRNAs than the other viruses infecting the plant.

### 3.8. Virus Detection in Fields

Forty-one of 186 tested trees had FYD symptoms, while 145 trees were asymptomatic. In the 41 symptomatic samples, the prevalent virus was GSPNeV (82.9%), followed by GSPEV (65.9%), GSPIV (19.5%) and GSPNuV (17.1%) (Table 2). In addition, a single infection of GSPNeV in FYD-affected plants was found in eight trees in four different areas surveyed, and it was the only virus detected in the Changshou orchard. Interestingly, in the four trees for which both asymptomatic and symptomatic branches were tested, GSPNeV was always detected in the 12 symptomatic branches with three for each tree combined in single detection, and not the asymptomatic branches (Table 2). These suggest an association between GSPNeV and the symptoms. However, six symptomatic samples were technically detected to be free of all the viruses (Table 2). In this case, the sensitivity of RT-PCR whereby fractional errors generally occur, or virus sequence variation which hampers primer binding might affect the test results. It is also possible that viruses are not evenly distributed throughout branches and therefore in some cases the sampled section did not contain virus. Alternatively, it is possible that some unknown virus or virus-like pathogens are responsible for the FYD.

The detection rate of GSPEV in 145 asymptomatic samples was the highest (33.1%), followed by GSPNEV (26.1%), GSPNuV (2.8%) and GSPIV (0.6%). Although we detected a frequent infection of GSPNeV in the symptomatic samples, 40 trees infected with this virus were found asymptomatic (Table S3). However, the results also showed that GSPNeV was not detected in the Yubei orchard where all trees were asymptomatic. In conclusion, regardless of the asymptomatic samples infected with GSPNeV, all together, the results of the etiology study including a virome analysis of a single tree revealed GSPNeV as the dominant virus in diseased trees, and therefore this virus might be the causal agent of the FYD of green Sichuan pepper. 

### 3.9. Graft and Natural Transmissible

Three months after graft inoculation, amplicons of expected sizes of all four viruses were obtained from all five seedlings grafted with the FYD-affected materials, not from the seedlings grafted with the healthy materials. The amplicons of each of the four viruses were cloned and sequenced, and sequence analysis confirmed the presence of the four viruses. The result shows that the viruses are graft transmissible. Whether there is an interaction between them in the transmission is unclear. GSPNeV is transmissible alone since it was detected in the stocks grated with the buds containing only this virus. However, further experiments are required to support that the other three viruses are capable of single transmission by grafting. In addition, no symptoms have yet developed on all these grafted stocks.

For the 31 seedlings sampled for the natural transmission experiment, GSPEV, GSPNeV, GSPNuV and GSPIV were detected in ten, six, one and one of them, showing existence of these viruses in the Ciyun breeding base. These plants were directly grown from seeds (as opposed to grafts) in open fields, and the evidence suggests that these viruses are naturally disseminated by either seeds or biological vectors, or both.

## 4. Discussion

The HTS, also known as next-generation sequencing and deep sequencing, is a sensitive and reliable technique to discover previously unknown viruses and study the virome of agricultural crops [46,47]. The options of enriching the sample for the viral nucleic acids, using virions extracts, total nucleic acids, dsRNA, poly-A RNAs, and small RNAs, among others, are available for viral diagnostics. Sequencing of small RNAs plus total RNA simultaneously satisfies convenience, encompassing all sequence information of viral pathogens (including viriods) [19]. For RNA-seq, the RNAs in library including those of virus origin are randomly fragmented to smaller sizes before synthesis of cDNA and addition of adaptors, and sequenced. By contrast, sRNAs of different sizes that are generated during plant RNA silencing against virus infection are produced by an endogenous silencing machinery [48,49,50]. Characteristics of virus-derived small interfering RNAs (vsiRNAs) provide insight into the interaction between a virus and its host plant. RNA-seq is more appropriate for the detection of some viruses with low titer in a plant than sRNA-seq, due to longer contigs from assembly of the RNA reads [51]. Combining RNA-seq, sRNA-seq, RT-PCR cloning and Sanger sequencing techniques, we discovered four novel RNA viruses belonging to distinct genera in the FYD-affected green Sichuan pepper tree in this study. However, the existence of other underlying viral pathogens without any sequence similarity to any known viruses is not entirely excluded.

Mix infection of four different RNA viruses in a single plant species supports the commonness of horizontal virus transfer. All four viruses are novel although each of them is distantly related to a group of known viruses in the established taxonomy system. Members of *Nepovirus* (*Comovirinae*, *Secoviridae*, *Picornavirales*) are bipartite viruses with positive-sense, single-stranded (+ss) RNA genome. Both RNA1 (~7.5 kb) and RNA2 (~3.9 kb) have a VPg linked to their 5’ end and poly (A) tail at their 3’ end, and each contains a ORF encoding a polyprotein [24]. Viruses of the genus *Idaeovirus* are composed of linear +ss RNA1 (5.4 kb), RNA2 (2.2 kb), and sgRNA3 (1.0 kb), that express replicase, MP and CP, respectively. They may have a stem-loop structure in the 3′ end of the genome that is not polyadenylated [29,52]. The genus *Enamovirus* (*Luteoviridae*) is yet to be assigned to an order. The genome of the monopartite viruses containing five ORFs is a linear, +ss RNA of 5.7 kb with a 5’-end VPg but without 3’ poly (A) tail [53]. The genus *Nucleorhabdovirus* (*Rhabdoviridae*, *Mononegavirales*) is characterized by having a liner negative sense (−) ssRNA genome (11–15 kb) encoding five to seven proteins in the order of 3’l-N-P-P3-M-G-L-5’t [54]. The results of comprehensive analyses and phylogenetics of the genomic sequences of the four novel viruses determined in this study are consistent with the salient nature described for known viruses. Therefore, they are provisionally named as GSPNeV, GSPIV, GSPEV and GSPNuV. The genome and encoded proteins of these viruses are divergent from known viruses infecting other plant species, suggesting they are distinct members of the genera *Nepovirus*, *Idaeovirus*, *Enamovirus* and *Nucleorhabdovirus*, respectively.

Virome analysis of the diseased tree revealed viral population profile and antiviral RNA silencing response by the host to the virus infections. High viral RNA read numbers from RNA-seq correlate with high levels of viral RNAs of genome, replication intermediate, or mRNA present during the infection, reproduction, or transcription in the diseased tissues. From this prospective, the high abundance of GSPNeV and GSPIV may suggest that these viruses replicate to higher titers than GSPEV and GSPNuV, or alternatively sampling may have occurred during active replication for these viruses. The green Sichuan pepper’s RNA silencing response to virus infection resembles that previously observed in other plants [55], as they show similar vsiRNAs characteristics. Although all the viruses are potentially affected by this same silencing system, it appears that GSPNeV is more easily targeted because it gave rise to much more vsiRNAs than those of other viruses. Intriguingly, in a comparative analysis the value dividing the transcriptome reads proportion by the sRNA reads proportion of GSPIV is 6.09 (5.3%/0.87%) much higher than 0.68 (7.15%/10.42%) of GSPNeV, 0.53 (0.39%/0.73%) of GSPEV, and 0.45 (0.05%/0.11%) of GSPNuV. It is likely that for some unknown reasons the other viruses have a general pattern of living in the plant according to the values (<1), whereas GSPIV not (>6). All these indications suggest that interactions of the plant and the viruses are complex.

The virus sequences obtained from the HTS forms the basis for unraveling the etiology of the viral diseases [56,57,58]. Specific, sensitive and rapid RT-PCR assays were developed and used to detect the viruses. The prevalence of GSPNeV and GSPEV is probably due to effective transmission of them by some vectors in the fields. It is interesting that apparently GSPIV has the highest viral copies in the sequenced plant (RNA2). A similar case has been found that a synergistic effect probably occurs when an idaeovirus coinfects with other viruses in the same plant [59]. However, the distribution of GSPIV in the field is very limited. Most of the diseased trees were found to be co-infected by GSPNeV and GSPEV in the surveyed growth regions. It is not clear whether one of them or both are causal agents of the FYD. However, many observations, without regard to whether all the FYD-infected trees were positive for GSPNeV in RT-PCR analysis, showed evidence to suggest that it might be the pathogen causing the FYD. Many asymptomatic samples were infected by GSPNeV, though this could be explained by clinical latency. If a pathogenic virus accumulates at low level in the plant, the infected plant is likely to be asymptomatic [60]. Taken this into consideration, we speculated that virus population of GSPNeV in the asymptomatic tree is small, and that the symptoms will be macroscopic once the virus titer reaches a high level over the course of a few years. It should be reminded that the asymptomatic trees infected with GSPNeV might be cultivars that are to some extent resistant to the virus infection, which is the rule rather than the exception for plant viruses [61]. Moreover, those viruses may represent the virulence-deficient variants, which do not induce symptoms. It is visible that the development of the FYD in single tree or among trees is a gradual course by time, and possible that initial manifestation of the FYD is showing no symptom. Therefore, we have inoculated the virus-free trees of disease-sensitive cultivar with GSPNeV-infected materials in order to fulfill Koch’s Postulates. Further observation of symptom expression is still ongoing.

The transmission of these viruses by grafting was proven in this study, and we have yet not observed any symptoms on grafted trees similar to those noted on the source tree. In fact, field development of FYD has been empirically observed as slow but suddenly destructive, and whether it has a latent period in asymptomatic trees is unclear [62]. Grafting, which is easy to implement, has become a common practice to propagate green Sichuan pepper trees, and will inevitably accelerate the distribution of the viral pathogens if the source trees are not certified. In addition, widespread of the viruses in the fields and breeding base suggests some natural dispersal of these viruses, other than those linked to production practices such as cutting and grafting. As previously reported, nepoviruses can be vectored by nematodes, pollens and seeds, and, in some cases, by mites and thrips [24]. Transmission of idaeoviruses by pollens and seeds could be rapid, but no biological vector has been reported [63]. Enamoviruses are transmitted by aphids [64], and nucleorhabdoviruses by insect species such as leafhoppers, planthoppers and aphids [54,65]. However, we have not addressed these transmissions in our study.

Farmers recently reported that the incidences of FYD have increased rapidly. As more and more trees are being planted, the viruses will severely jeopardize the sustainability of green Sichuan pepper production. At the current moment, we are focusing on investigating the role of each of the four viruses on FYD. Preliminary observations noted various symptoms, which might be associated with different viruses in the plants. However, a further test is necessary to confirm the associations for solid conclusions. Considering again the natural infections of these viruses in seedlings, future work will focus on elucidating the mechanism of viral transmission and developing a virus-free certificate program to distribute seedlings to farmers.

In summary, we identified four new graft- and naturally-transmissible RNA viruses by HTS of RNA- and sRNA-seq techniques, obtained the complete viral genomes based on cloning and Sanger sequencing, and investigated their occurrence in several major producing regions in Chongqing, using RT-PCR. Filed survey allows us to find the association of GSPNeV with the FYD. The virome analysis also suggests such an association. Further characterization of the viral sRNA populations recalls a typical virus-plant interaction model with respect to RNA silencing, thus an important mechanism could be utilized in combating against viral infections in plants [66,67,68]. Future works of urgent need were discussed. All together these data provide significant information for virus control as well as disease management, contributing to the sustainability of green Sichuan pepper production.

## Figures and Tables

**Table 1:** Analysis of high-throughput sequencing data from the diseased tissues of green Sichuan pepper affected by the flower yellowing disease.

Viruses	Contigs Amount	Contigs Size (bp)	Viral Reads	% of Total Reads	Average Coverage
*[RNA-seq (RNA1*&*RNA2)]|[sRNA-seq (RNA1*&*RNA2)]*					
GSPNeV	2|12	6,945&6,170|145–391	4,152,530|3,050,449	7.15|10.42	34,717&49,160|3,502&5,164
GSPIV	2|40	5,433&2,372|58–241	3,078,099|254,692	5.3|0.87	24,850&145,377|532&2,515
GSPEV	1|11	4,729|83–152	226,501|213,707	0.39|0.73	6,129|784
GSPNuV	1|16	13,548|67–139	29,038|32,202	0.05|0.11	405|52

**Table 2:** Detection of viruses in the FYD-affected green Sichuan pepper trees collected from Chongqing Province.

Locations		Samples	Idaeovirus	Nepovirus	Nucleorhabdovirus	Enamovirus
Bishan county	Fulu Town	4	−	+	−	+
		1	−	−	−	−
		1*	−	+	−	−
Jiangjin district	Xianfeng Town	1	−	+	+	+
		3 ^1^	−	+	−	+
		1	−	−	+	+
	Ciyun Town	6 ^2^	−	+	−	+
		3*	−	+	−	−
		2	−	−	−	−
	Jiangjin district	3	+	+	+	+
		2	−	+	+	+
		5	+	+	−	+
		2	−	+	−	+
		1*^1^	−	+	−	−
Changshou district	Dandu Town	3	−	−	−	−
		3*	−	+	−	−
**Total**		41	8	34	7	27
**Detection rate**			19.5%	82.9%	17.1%	65.9%
**Single virus infection**			0	8	0	0

* Single virus infected sample. ^1^ and ^2^: number of trees which had both symptomatic and asymptomatic branches (results shown are from symptomatic branches).

## Supplementary Materials

The following are available online at https://www.mdpi.com/1999-4915/11/8/696/s1. Figure S1: The size distribution of total small RNAs from symptomatic green Sichuan pepper sample. Table S1: List of primers used in this study. Table S2: The RT-PCR reaction systems and running programs of viruses detection. Table S3: Detection of the viruses in the asymptomatic green Sichuan pepper trees collected from Chongqing Province.
