# Supplementary material for: Discovery of Four Novel Viruses Associated with Flower Yellowing Disease of Green Sichuan Pepper (Zanthoxylum armatum) by Virome Analysis
Source: Viruses. 2019 Jul 31;11(8):696. doi: 10.3390/v11080696 (PMC6723833; doi:10.3390/v11080696)
Supplement: Supplementary file 1 [file viruses-11-00696-s001.zip › viruses-539649 - supplementary/Table S1.docx]

**Table S1**. List of primers used in this study

| **Contigs primers** | | | **Sequence (5' to 3')** | | **Base (nt)** | **Size (bp)** |
| --- | --- | --- | --- | --- | --- | --- |
| **ZPNeV_RNA1** | | 1F-169 | | CACATTGTTGAAGTTCGTGC | 20 | 1562 |
|  | | 1R-1712 | | ATGCCCTCCATTGACTGAC | 19 |  |
|  | | 2F-1230 | | TTCCTACGCTTCTGCTAC | 18 | 1966 |
|  | | 2R-3178 | | GGTGACAAGACCACTCAA | 18 |  |
|  | | 3F-2378 | | TGGCTGATGTGGAGAGTA | 18 | 1916 |
|  | | 3R-4276 | | GATGAGAGCAATGGTAGC | 18 |  |
|  | | 4F-4095 | | AAGTGATGGTAGCGTGTC | 18 | 1651 |
|  | | 4R-5726 | | CTTGTAGTAATGAAGGCAGG | 20 |  |
|  | | 5F-4991 | | CGATTTGTCCCTTTCCTTAC | 20 | 2578 |
|  | | 5R-6651 | | GCTTACACATTCGCCCAT | 18 |  |
| **ZPNeV_RNA2** | | 1F-490 | | TCTCTCCCACCTAAGCCTA | 19 | 1782 |
|  | | 1R-2254 | | GAAGTCAATAAGACCGCC | 18 |  |
|  | | 2F-1543 | | GATGAAGAAGGATGCGAC | 18 | 1792 |
|  | | 2R-3317 | | GGGAGAAGATTTGCTGTG | 18 |  |
|  | | 3F-2932 | | GAGGAGACATTCAAGCGAA | 19 | 1730 |
|  | | 3R-4644 | | TGCCTTTCATCAGTGGTG | 18 |  |
|  | | 4F-3900 | | TTGCGGACAACTTACCGT | 18 | 1984 |
|  | | 4R-5866 | | ATGCTTACACATTCGCCC | 18 |  |
| **ZPIV_RNA1** | | 1F-163 | | ATTGTGACGCTATCGCCT | 18 | 1538 |
|  | | 1R-1683 | | ATCCACGAGTTTCCGAGA | 18 |  |
|  | | 2F-1221 | | CTTGATAAACTTGTGGGTGG | 20 | 1868 |
|  | | 2R-3070 | | ATGACTGTGATGTCCTGGG | 19 |  |
|  | | 3F-2971 | | TCTGTAATAGAGTCGCCG | 18 | 1763 |
|  | | 3R-4715 | | GTCACCTCCGATAAGGATA | 19 |  |
|  | | 4F-3819 | | CAGGTTGGTTCTATCAGATG | 20 | 1590 |
|  | | 4R-5391 | | TAACGAGAGGTTTAGCCC | 18 |  |
| **ZPIV_RNA2** | | 1F-622 | | TAAGGATAGGTCAGCCATC | 19 | 1567 |
|  | | 1R-2172 | | ATAAAGAGGTAAGCCCGC | 18 |  |
| **ZPEV_RNA** | | 1F-871 | | CTTCAAATCAATAGTCCGCCTTCAG | 25 | 1958 |
|  | | 1R-2804 | | TCTACCACACCATCAATCACAGCCT | 25 |  |
|  | | 2F-2798 | | CAGTTCAGGCTGTGATTGATGGTGT | 25 | 2151 |
|  | | 2R-4927 | | GGATGTCGGCTGAATACGTGAAGTC | 25 |  |
| **ZPNuV_ RNA** | | 1F-454 | | AGAGCGAACTCACATAGCCC | 20 | 2292 |
|  | | 1R-2728 | | ACTTCTCGGATGGCGTTA | 18 |  |
|  | | 2F-1459 | | AAGAAATCTCCGAGTCTACGCT | 22 | 2788 |
|  | | 2R-4228 | | AACTAATCGGAGGAGGCGA | 19 |  |
|  | | 3F-4214 | | TTAGGACTTGATTGTCGCC | 19 | 1938 |
|  | | 3R-6132 | | CATTTGATAGATGGTCACGC | 20 |  |
|  | | 4F-4829 | | AGAGGTAATCTTTCAGGCGA | 20 | 2421 |
|  | | 4R-7230 | | TGGGATGCCACTAAGGACTA | 20 |  |
|  | | 5F-6955 | | CCACTGCCATTGTCGTTA | 18 | 1900 |
|  | | 5R-8836 | | TGTGCTGCTGAAGAAACGG | 19 |  |
|  | | 6F-8743 | | AGCCATCTCCTTACCTTGCG | 20 | 2018 |
|  | | 6R-10739 | | TACCACCCTCTCCGACACTTAG | 22 |  |
|  | | 7F-10724 | | TGGGTGGGAGTAATGCTAAGT | 21 | 2385 |
|  | | 7R-13088 | | TGTATGAGATGCTATCCGCTG | 21 |  |
| **RACE primers** | | | **Sequence (5' to 3')** | | **Base (nt)** | **Size (bp)** |
| **ZPNeV_RNA1** | 5R-GSP-270 | | GCGGATCGCCTTCTTCTCCTTAATG | | 25 | 294 |
|  | 5R-GSP-380 | | GCTTCAGCCATCTTCTTCTTCTCCT | | 25 | 404 |
|  | 3R-GSP-6518 | | CCTCTTCTCCCTCTTTCCTAATTGA | | 25 | 1354 |
|  | 3R-GSP-6401 | | CTGGATGCTCCGATCTTTCTTATTA | | 25 | 1471 |
| **ZPNeV_RNA2** | 5R-GSP-390 | | GATTTGCGTTCTCTCAGCTCTCTTC | | 25 | 414 |
|  | 5R-GSP-515 | | CGATACTTGGCGAGGAGACATTGAG | | 25 | 539 |
|  | 3R-GSP-5733 | | TAAGGGATTCTCTTTCCTAATTGGG | | 25 | 1351 |
|  | 3R-GSP-5630 | | TTATTATTTGAGTATCCCCTCCCTT | | 25 | 1454 |
| **ZPIV_RNA1** | 5R-GSP-459 | | GATCATCGAACTCTGACGAGAACAC | | 25 | 483 |
|  | 5R-GSP-614 | | ATCATCATCACACAGTTTTTGGGGA | | 25 | 638 |
|  | 3R-GSP-4590 | | AAAGAGATACGCACGGGTTTAGCAG | | 25 | 498 |
|  | 3R-GSP-5053 | | CGCCTAGTTTATATGATGCCTTGTG | | 25 | 395 |
| **ZPIV_RNA2** | 5R-GSP-223 | | TCTGACCATCTTGTCCTTCTTTCTG | | 25 | 247 |
|  | 5R-GSP-339 | | GTTAGTGAAGAAGGCTTAGTGGGGA | | 25 | 363 |
|  | 3R-GSP-1871 | | TTGCAGTCCTTGAGAAAGACGCTAC | | 25 | 364 |
|  | 3R-GSP-1788 | | CGTTCAGCGACAGGGTGATAAGTTG | | 25 | 467 |
| **ZPIV_sgRNA3** | 5R-GSP-194 | | ACTGAGCTTTAACTGTTGGAGGAAT | | 25 | 218 |
| **ZPEV_RNA** | 5R-GSP-768 | | AACAGACATAAAGTCCCCACCAACG | | 25 | 792 |
|  | 5R-GSP-824 | | GACTCATCAGGAGCCTCAGGAAGAA | | 25 | 848 |
|  | 3R-GSP-5063 | | CAGTTTCTGTGACGATTGATGAGGT | | 25 | 525 |
| **ZPNuV_RNA** | 5R-GSP-319 | | ATACTGCATTGCATAGTGCGTCATA | | 25 | 343 |
|  | 5R-GSP-447 | | GCTATGTGAGTTCGCTCTGAAATCT | | 25 | 471 |
|  | 3R-GSP-13228 | | ATATATTCTCTGTGGCTGCACTGGC | | 25 | 319 |
|  | 3R-GSP-13083 | | GAGTGCAGCGGATAGCATCTCATAC | | 25 | 464 |
| **Detection primers** | | | **Sequence (5' to 3')** | | **Base (nt)** | **Size (bp)** |
| **ZPNeV_RNA2** | Nep-R2-3093df | | CGCAGGCTTTGATTGAGAC | | 19 | 717 |
|  | Nep-R2-3809dr | | AAACACGACCTCCACGCTT | | 19 |  |
| **ZPIV_RNA2** | Idea-R2-1580df | | GGAGGAAATCTTGGGAAAC | | 19 | 710 |
|  | Idea-R2-2307dr | | GCAACACGACCTTTATCG | | 18 |  |
| **ZPEV_RNA** | Ena3dr | | GGTTTCAATGAGGTAGGCG | | 19 | 483 |
|  | Ena3df | | GGGAGGACACCCTGAGATAA | | 20 |  |
| **ZPNuV_RNA** | Nuc-9881df | | TGCCCACATCCTCTTTAGAC | | 20 | 890 |
|  | Nuc-10770dr | | TACCACCCTCTCCGACACTTAG | | 22 |  |
