# Supplementary material for: Discovery of Four Novel Viruses Associated with Flower Yellowing Disease of Green Sichuan Pepper (Zanthoxylum armatum) by Virome Analysis
Source: Viruses. 2019 Jul 31;11(8):696. doi: 10.3390/v11080696 (PMC6723833; doi:10.3390/v11080696)
Supplement: Supplementary file 1 [file viruses-11-00696-s001.zip › viruses-539649 - supplementary/Table S2.docx]

**Table S2.** RT-PCR reaction systems and running programs of the viruses detection

| **Primer name** | **Reaction system** | | | | | **RT-PCR program** | | | | | |
| --- | --- | --- | --- | --- | --- | --- | --- | --- | --- | --- | --- |
|  | RNA extracts | 2X buffer | primers | RT/Taq | ddH20 | reverse transcription | pre-denaturation | 40 cycles | | | final  extension |
|  |  |  |  |  |  |  |  | denaturation | annealing | extension |  |
| Nep-R2-3093df/3809dr | 1 ul | 10 ul | 1.2 ul | 0.5 ul | 8 ul | 50℃,  30 min | 94℃,  2 min | 94℃, 30s | 53℃,  30 s | 72℃,  1 min | 72℃,  10 min |
| Nuc-9881df/10770dr |  |  |  |  |  |  |  |  |  |  |  |
| Idea-R2-1580df/2307dr |  |  |  |  |  |  |  |  |  |  |  |
| Ena3dr/3df | 1 ul | 12.5 ul | 2 ul | 0.5 ul | 10 ul | 50℃,  30 min | 94℃,  2 min | 94℃,  30s | 53℃,  30 s | 72℃,  30s | 72℃,  10 min |
