# Supplementary material for: Discovery of Four Novel Viruses Associated with Flower Yellowing Disease of Green Sichuan Pepper (Zanthoxylum armatum) by Virome Analysis
Source: Viruses. 2019 Jul 31;11(8):696. doi: 10.3390/v11080696 (PMC6723833; doi:10.3390/v11080696)
Supplement: Supplementary file 1 [file viruses-11-00696-s001.zip › viruses-539649 - supplementary/Table S3.docx]

**Table S3.** Detection of the viruses in asymptomatic Zhuye pepper trees collected from Chongqing province

| **Items** | | | **Total samples** | **Positive samples** | **Idaeovirus** | **Nepovirus** | **Nucleorhabdovirus** | **Enamovirus** |
| --- | --- | --- | --- | --- | --- | --- | --- | --- |
| **Locations** | Bishan County | Fulu | 18 | 18 | 0 | 18 | 0 | 8 |
|  |  | Guangpu | 25 | 21 | 0 | 16 | 2 | 14 |
|  |  | Sanhe | 18 | 1 | 0 | 1 | 0 | 0 |
|  | Jiangjin District | Xianfeng | 18 | 2 | 0 | 2 | 2 | 16 |
|  |  | Ciyun | 26 | 7 | 0 | 6 | 0 | 8 |
|  | Yubei District | Shichuan | 20 | 1 | 0 | 0 | 0 | 1 |
|  | Changshou District | Dandu | 20 | 0 | 0 | 0 | 0 | 0 |
| **Total** | | | 145 | 64 | 0 | 40 | 4 | 47 |
| **Detection rate** | | | | | 0 | 27.6% | 2.7% | 33.1% |
| **Single virus infection** | | | | | 0 | 14 | 1 | 18 |
