# Supplementary figures and images for: Discovery of Four Novel Viruses Associated with Flower Yellowing Disease of Green Sichuan Pepper (Zanthoxylum armatum) by Virome Analysis
Source: Viruses. 2019 Jul 31;11(8):696. doi: 10.3390/v11080696 (PMC6723833; doi:10.3390/v11080696)

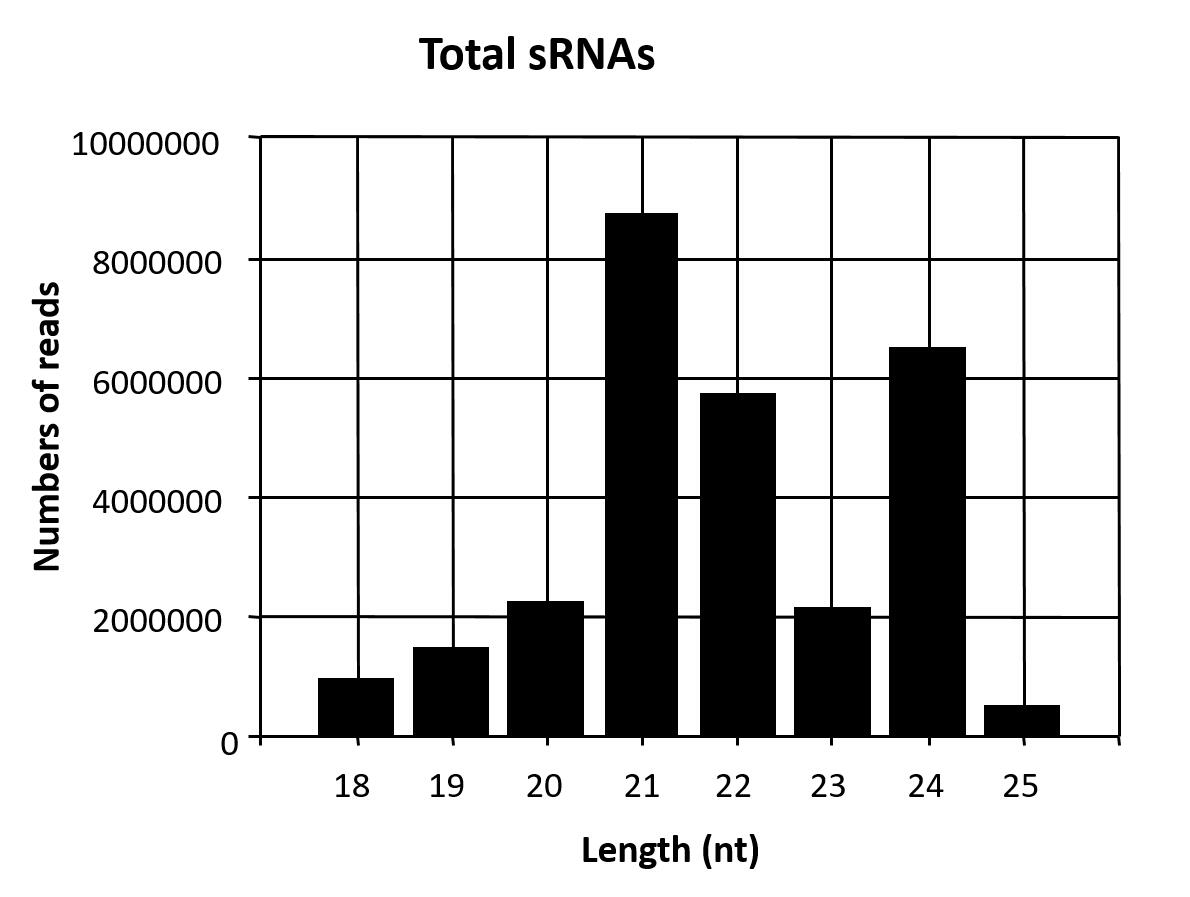

Supplement: Supplementary file 1 [file viruses-11-00696-s001.zip › viruses-539649 - supplementary/Figure S1.jpg]
